# Nanoplastic Exposure Impairs Host Fitness and Parasite Transmission With Transgenerational Maternal Reproductive Costs in the *Daphnia*‐Fungal Parasite System

**DOI:** 10.1002/ece3.74591

**Published:** 2026-10-28

**Authors:** Vanderville Villegas, Lilith Biere, Kristel F. Sánchez, Justyna Wolinska

**Affiliations:** ^1^ Leibniz Institute of Freshwater Ecology and Inland Fisheries Berlin Germany; ^2^ Institute of Biology Freie Universität Berlin Berlin Germany

**Keywords:** *Australozyma monospora*, host–parasite interactions, maternal effects, *Metschnikowia bicuspidata*

## Abstract

Nanoplastics are emerging contaminants in aquatic ecosystems, but their direct and maternally transmitted effects on host–parasite interactions remain poorly understood. We investigated the effects of polystyrene nanoplastic exposure (50 nm, 5 mg L^−1^) on *Daphnia magna* infected with the fungal parasite *Australozyma monospora* across two host generations. Direct nanoplastic exposure occurred in the parental generation (F0), whereas F1 offspring were raised in nanoplastic‐free medium to isolate maternal nanoplastic effects. Direct nanoplastic exposure severely impaired F0 host fitness, reduced reproductive probability and total offspring production, while halving median survival time relative to controls. Nanoplastic exposure also impaired parasite maturation likelihood by decreasing host survival prior to transmission. Infection prevalence and spore production did not differ between nanoplastic‐exposed and control hosts among individuals that survived to the transmission stage, suggesting that nanoplastic exposure compromises parasite fitness primarily through accelerated host mortality rather than through within‐host suppression. In the F1 generation, offspring of nanoplastic‐exposed mothers had lower reproductive probability and delayed age at first clutch, despite experiencing no direct nanoplastic exposure. Host survival and parasite fitness outcomes were not altered in F1. Together, these results demonstrate that nanoplastic pollution disrupts host–parasite dynamics both directly and across generations, imposing reproductive costs that persist in unexposed offspring through maternal effects. Such transgenerational effects compromise host population recovery and amplify parasite transmission in nanoplastic‐contaminated freshwater environments.

## Graphical abstract

Polystyrene nanoplastics (50 nm) severely reduced the fitness of *Daphnia magna* infected with the fungal parasite *Australozyma monospora*, halving parental survival and suppressing reproduction, and they lowered parasite maturation indirectly by killing hosts before transmission rather than by limiting within‐host infection. Offspring of exposed mothers inherited reproductive costs, showing reduced reproductive probability and delayed first reproduction despite never encountering nanoplastics directly. These findings show that nanoplastic pollution disrupts host‐parasite dynamics within and across generations, with transgenerational reproductive costs that could impair host population recovery in contaminated freshwater ecosystems.

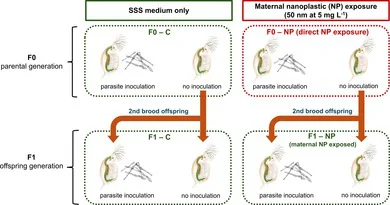

## 1 Introduction

Plastic pollution has emerged as a pervasive and escalating threat to freshwater ecosystems, raising urgent concerns about its ecological consequences. Once in aquatic environments, plastics fragment into microplastics (< 5 mm) (Arthur et al. 2009) and nanoplastics (< 1000 nm) (Gigault et al. 2021) through UV weathering and mechanical abrasion (Atugoda et al. 2022). These particles are now ubiquitous from remote lakes to polar ice (Materić, Kjær, et al. 2022; Materić, Peacock, et al. 2022). Nanoplastics are of particular concern due to their small size, high surface reactivity and capacity to cross biological barriers (Gigault et al. 2021), inducing oxidative stress and inflammatory responses in exposed organisms (Tang 2025). Experimental studies show that nanoplastic ingestion negatively affects the fitness of microbes, algae, invertebrates and fish (reviewed in Funke et al. 2024; Shi et al. 2024), and that nanoplastics can be transferred across trophic levels within aquatic food webs (Atugoda et al. 2022; Mattsson et al. 2017). Recent work shows that nanoplastic exposure increases *Daphnia* (Crustacea: Cladocera) susceptibility to fungal parasitism and enhances parasite proliferation within the host (Manzi et al. 2023; Mavrianos et al. 2023; Webb et al. 2025), through an indirect pathway involving nanoplastic‐mediated disruption of the host microbiome (Villegas et al. 2025, 2026). Despite growing evidence that nanoplastics can modify host–parasite interactions (reviewed in Balsdon and Koprivnikar 2024), whether these effects extend across generations through maternal inheritance remains unknown.

Maternal effects, defined as non‐genetic influences of the maternal environment on offspring phenotype, are well documented in water fleas of the genus *Daphnia* and can arise through altered resource provisioning, epigenetic modifications, or the direct transfer of contaminants to eggs (reviewed in Agrelius and Dudycha 2025; Mousseau and Fox 1998). Maternal exposure to nanoplastics may represent an ecologically relevant scenario. Although nanoplastic pollution is often chronic, spatial heterogeneity and particle dispersal may result in *Daphnia* offspring experiencing different exposure levels than their mothers (Di et al. 2024; Gündoğdu et al. 2026). Nanoplastics can be transferred from *Daphnia* mothers to offspring via the brood chamber and gut‐ovary‐oocyte pathways (Xue et al. 2023), and their accumulation has been linked to transgenerational reproductive impairment (Liao et al. 2023). Maternal nanoplastic exposure has also been shown to suppress offspring development in zebrafish through mitochondrial dysfunction (Cao et al. 2025). While both direct and maternal nanoplastic exposure were shown to impair organismal traits such as survival, reproduction and immune function (Liu et al. 2020; Villegas et al. 2026), their consequences for ecological interactions such as host–parasite dynamics remain poorly understood. In particular, whether maternal nanoplastic exposure alone modifies offspring susceptibility to parasitic infection has received little attention, leaving a critical gap in our understanding of how nanoplastic pollution influences host–parasite systems across generations.

The *Daphnia magna–Australozyma monospora* sp. nov. (formerly *Metschnikowia bicuspidata* , Lachance et al. 2025) host–parasite system provides a tractable model for addressing this gap. *Daphnia magna* Straus (1820) is a keystone freshwater consumer widely used in ecotoxicology and evolutionary ecology due to its clonal reproduction, short generation time and sensitivity to environmental stressors (Altshuler et al. 2011; Ebert 2022). *Australozyma monospora* is a common and virulent fungal parasite infecting natural *Daphnia* populations: hosts ingest waterborne spores, which penetrate the gut wall, proliferate in the haemolymph and reduce host fecundity and lifespan (Cáceres et al. 2006; Stewart Merrill and Cáceres 2018). The clonal nature of *Daphnia* allows us to expose parental mothers to nanoplastics, then raise their succeeding generations of offspring in nanoplastic‐free conditions, cleanly isolating maternal nanoplastic legacy from direct offspring exposure.

Here, we investigated the direct and maternal effects of nanoplastic exposure on host and parasite fitness in the *Daphnia magna‐Australozyma monospora* host–parasite system. Specifically, we aimed to (i) determine how direct nanoplastic exposure affects host fitness across reproductive and survival traits, (ii) assess whether nanoplastic exposure alters host mortality and parasite fitness following parasite challenge, (iii) determine whether maternal nanoplastic exposure produces transgenerational effects on offspring fitness, and (iv) test whether parasite‐related effects in the parental generation persist into the F1 generation through maternal carry‐over effect.

To address these objectives, we formulated directional hypotheses. First, we expected direct nanoplastic exposure to reduce host fitness across reproductive and survival traits. Second, we predicted that nanoplastic exposure would increase host mortality following parasite challenge, thereby reducing the time available for parasite development within infected hosts. However, among hosts surviving to the stage of parasite transmission, we expected nanoplastic exposure to increase infection prevalence and spore production, consistent with reports on nanoplastic‐induced enhancement of parasite fitness (Manzi et al. 2023; Mavrianos et al. 2023; Webb et al. 2025). Third, we expected maternal nanoplastic exposure to affect offspring fitness, particularly reproductive traits. Finally, we hypothesised that parasite‐related effects observed in the parental generation could partially persist into the F1 generation through maternal carry‐over effects. By integrating host life‐history traits with parasite infection dynamics across two generations, this study provides a comprehensive assessment of how nanoplastic exposure, including maternal exposure, can shape host–parasite interactions and potentially influence disease dynamics in nanoplastic‐contaminated freshwater environments.

## 2 Materials and Methods

### 2.1 Host–Parasite System

*Daphnia magna* (genotype E17:07), originally isolated from a temporary pond near Oxford, England (Hesse et al. 2012), was used due to its high susceptibility to *Australozyma monospora* (Manzi et al. 2023). This genotype has also been employed in previous studies on host–parasite (Webb et al. 2025) and host‐microbiome responses to nanoplastics (Villegas et al. 2025, 2026). Stock cultures were maintained in glass jars containing 150 mL modified SSS medium (artificial *Daphnia* medium; Saebelfeld et al. 2017) at 19°C under a 14:10 h light: dark photoperiod. Animals were fed three times per week with the green alga *Tetradesmus obliquus* at a concentration of 1 mg C L^−1^. *T. obliquus* was cultured in modified Z‐medium (Zehnder and Gorham 1960) at 19°C under a 14:10 h light: dark photoperiod and harvested biweekly. *Daphnia* medium was renewed every 4 days, and cultures were maintained until synchronised third‐clutch offspring were produced for use in the experiment.

The fungal parasite *A*. *monospora* strain ‘VRANOV_2018’, isolated in 2018 from the Vranov reservoir, Czech Republic, was maintained on *D. magna* E17:07. Infection begins when hosts ingest mature needle‐like spores (ascospore) enclosed in asci, which penetrate the gut epithelium and proliferate within the haemolymph (Stewart Merrill and Cáceres 2018). Successful infection is indicated by whitish regions in the host body cavity (Stewart Merrill and Cáceres 2018), typically observable 8–10 days post‐inoculation (Manzi et al. 2023).

### 2.2 Nanoplastic Suspension

Spherical 50 nm polystyrene nanoplastics (Micromod Partikeltechnologie GmbH, Germany; micromer‐greenF, product code: 29‐00‐501) were used. These plain, fluorescently labelled particles were suspended in distilled water without additives and stored at 4°C in the dark. Detailed particle characteristics and suspension stability are described in Villegas et al. (2025). A working suspension of 5 mg L^−1^ was prepared in SSS medium. This concentration was selected based on prior studies showing significant effects of nanoplastic exposure on *Daphnia‐Australozyma* interactions (Webb et al. 2025) and on the *Daphnia* microbiome (Villegas et al. 2025), with stronger effects observed for 50 nm particles at 5 mg L^−1^ compared with 100 nm and 1 mg L^−1^, respectively. Although the selected concentration exceeds the highest concentration reported in lentic freshwater systems (24–1588 μg L^−1^ total concentration, Materić, Peacock, et al. 2022), environmental nanoplastic exposure is expected to be spatially and temporally heterogeneous (Pradel et al. 2023), with elevated concentrations potentially occurring near point sources. Thus, the concentration represents a relatively high, but potentially relevant exposure scenario.

### 2.3 Experimental Design and Procedures

The experiment included eight treatment groups defined by generation (F0: maternal exposure; F1: offspring), nanoplastic (NP) treatment (control: SSS medium; NP: 50 nm, 5 mg L^−1^), and parasite treatment (inoculated vs. non‐inoculated) (Figure 1). Second‐clutch offspring from parasite non‐inoculated individuals from each F0 generation were used to establish the F1 generation. F1 individuals originating from each maternal nanoplastic treatment (control vs. NP) were assigned to the same parasite exposure design as the F0 generation but were maintained entirely in nanoplastic‐free medium. The term ‘F1‐NP’ refers to offspring derived from nanoplastic‐exposed mothers (F0‐NP), representing a maternal nanoplastic exposure effect, whereas ‘F1‐C’ refers to offspring derived from control mothers (F0‐C). Each treatment group contained 15 replicates, except for two F1 control groups, which contained 10 replicates due to limited availability of synchronised juveniles, resulting in a total of 110 experimental units.

**FIGURE 1:**
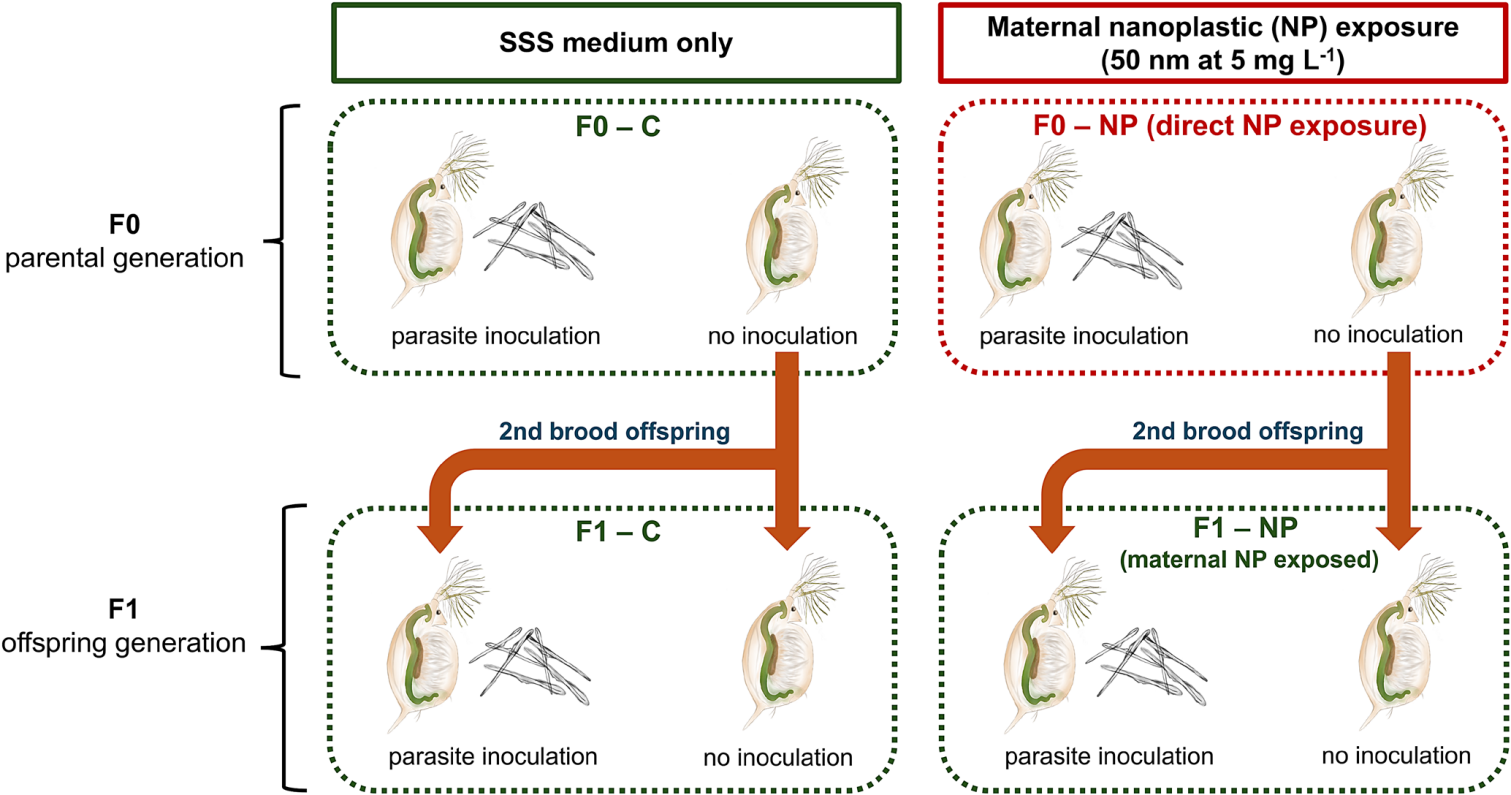
Schematic diagram of the experimental design testing maternal effects of nanoplastic (NP) exposure on *Daphnia–Australozyma* host–parasite system. F0 denotes the parental generation and F1 the offspring generation. In F0, animals were either exposed to nanoplastics (50 nm; 5 mg L^−1^) or maintained under control conditions (SSS medium only). Within each maternal treatment, individuals were inoculated either with *Australozyma* spores or with a sham inoculum (homogenised uninfected *Daphnia* tissue). Second‐brood F1 offspring were collected from sham‐inoculated (i.e., non‐inoculated) F0 mothers and then assigned to either parasite inoculation or sham exposure, following the same infection protocol as in the F0. All F1 individuals were subsequently maintained in control SSS medium throughout.

#### 2.3.1 Establishment of *Daphnia* F0 and F1 Generations and Parasite Infection

*Daphnia magna* female juveniles (< 24 h old; sexed using a stereomicroscope, Nikon SMZ800N, Japan) were individually transferred to 100 mL SSS medium (control) or nanoplastic suspension in experimental Day 1 and transferred again to 5 mL of fresh medium of the same treatment on Day 6, when parasite inoculation was performed (a smaller volume was used to concentrate parasite spores). Each inoculated host received approximately 3500 spores mL^−1^, consistent with prior studies (Manzi et al. 2023; Villegas et al. 2025, 2026; Webb et al. 2025). To prepare the inoculum, five highly infected *D. magna* were homogenised in 1 mL SSS medium, and mature ascospores were counted using an improved Neubauer chamber under 400× phase contrast microscopy (Carl Zeiss Axioscope, Germany). Non‐inoculated controls received homogenates from five uninfected hosts (sham inoculum). No feeding was performed on the day of parasite inoculation. After 48 h, individuals were transferred back to 100 mL spore‐free medium corresponding to their treatment. The F0 experiment concluded on Day 30.

F1 groups were established from second‐clutch juveniles (< 48 h old) produced by non‐inoculated F0 mothers and were maintained in nanoplastic‐free medium. Parasite infection in the F1 generation followed the same infection protocol used for F0. The F1 experiment also concluded on Day 30.

Both generations were maintained throughout the experiment at 19°C under a 12:12 h light: dark cycle, fed daily with 1 mg C L^−1^ *T. obliquus* (except on the inoculation day), and the medium was renewed every 4 days.

#### 2.3.2 *Daphnia* Rinsing to Prevent Generational Nanoplastic Carry‐Over

Nanoplastics can enter the brood chamber and accumulate in developing eggs (Xue et al. 2023). To minimise nanoplastic transfer and thereby isolate maternal effects, F0 mothers were rinsed prior to the release of the second clutch. Within 24 h after first clutch release, F0 mothers were transferred to 100 mL of nanoplastic‐free medium and maintained for an additional 24 h. Mothers were then transferred to fresh 100 mL nanoplastic‐free medium to prevent reuptake of egested particles and maintained there until the second clutch was released. Afterwards, mothers were returned to nanoplastic‐containing medium. This procedure allows most ingested nanoplastics to be egested within 24 h (Liu, Zhang, et al. 2024; Zhang et al. 2023). All experimental groups in both generations underwent the same rinsing protocol to prevent confounding effects.

#### 2.3.3 Reproduction, Mortality and Infection Monitoring

Reproduction and mortality were recorded daily. Offspring were collected, counted, sexed and discarded unless used to establish the F1 generation. Mortality was confirmed by the absence of a heartbeat. All parasite‐inoculated individuals were examined under a stereomicroscope at 400× magnification for signs of infection. Infection was confirmed by the presence of dark regions within the host body cavities, resulting from dense aggregations of parasite spores. Following death, infected hosts were homogenised and fixed in 5% formaldehyde and stored at 4°C until spore counting. By the end of the experiment (Day 30), all individuals with confirmed infections were dead.

### 2.4 Analysis of Host and Parasite Fitness Parameters

All analyses were conducted separately for the F0 and F1 generations to distinguish direct effects of nanoplastic exposure from maternal carry‐over effects. Statistical analyses were performed in R v4.5.2 (R Core Team 2023) using RStudio v.2025.09.2‐418 (Posit Team 2024). Visualisations were generated using the ggplot2 package (Wickham 2016). Survival was analysed using Kaplan–Meier (KM) survival curves with the survival package (Therneau et al. 2024), and visualised with survminer (Kassambara et al. 2026). Models were fitted using the glmmTMB package (Brooks et al. 2026). Model assumptions and fit were assessed using scaled randomised quantile residuals via the DHARMa package diagnostics for hierarchical regression models (Hartig 2024), with formal tests for overdispersion and zero inflation applied to each candidate model. Model selection followed an information‐theoretic framework (Anderson and Burnham 2002) based on Aikake's Information Criterion (AIC) and the Bayesian Information Criterion, with the best‐fitting model (lowest criterion value) retained for inference. These criteria weigh goodness of fit against model complexity by penalising the number of estimated parameters (Burnham and Anderson 2004).

#### 2.4.1 Host Fitness Parameters

All inoculated individuals were retained in the inoculated group and included in host fitness analyses, irrespective of infection success. Host fitness was assessed using total offspring production, age at first clutch (i.e., the age at which first‐clutch juveniles were born), reproductive probability (i.e., the proportion of individuals reproducing at least once) and host survival (i.e., the number of days, with survival time censored at the end of the experiment on Day 30).

Total offspring were analysed as count response variables. Variance‐to‐mean dispersion ratios ruled out Poisson models. Negative binomial GLMs (nbinom2 parameterisation) were fitted, with group‐specific dispersion structure (*dispformula* = ~ group * inoc) added. For age at first clutch, F0 was analysed using Gamma GLM with a log link provided better fit than Gaussian alternatives. For F1, given the near‐discrete clustering of reproductive timings, a Gaussian GLM with group‐specific dispersion was retained. Reproductive probability was modelled as a binary outcome using binomial GLMs with a logit link. For F0, the full interaction model (reproduced ~ group * inoc) was fitted. For F1, the full model produced complete separation arising from 100% reproduction in the control inoculated group, inflating standard errors. An additive model (reproduced ~ group + inoc) resolved the separation arising from 100% reproduction in the control inoculated group. Firth penalised likelihood regression using logistf package (Heinze et al. 2025) was applied as a robustness check.

Host survival was analysed using time‐to‐event methods. The experiment ran for 30 days; animals alive at the end were right‐censored at Day 30. Death was coded as the event of interest (event = 1), whereas individuals surviving to Day 30 were treated as right‐censored (event = 0). Kaplan–Meier survival curves were estimated per treatment group, and overall group differences assessed with the log‐rank test. Accelerated Failure Time (AFT) models with a Weibull distribution were fitted separately per generation to formally test the effects of nanoplastic treatment, inoculation and their interaction on survival time while accounting for censoring. Model‐estimated marginal means and 95% confidence intervals for all response variables were obtained using the emmeans package (Lenth et al. 2026), with back‐transformation from the link scale where applicable. Model adequacies were evaluated using residual diagnostics via the survival package (Therneau et al. 2024).

#### 2.4.2 Parasite Fitness Parameters

Pre‐transmission survival, defined as whether an inoculated host survived long enough for the parasite to complete development and reach the transmissible stage, was modelled as a binary outcome using binomial GLMs with a logit link. Infection prevalence was analysed in animals that survived to the pre‐transmission stage. For F0, the nanoplastic‐exposed survivor pool comprised only two animals, precluding statistical modelling; infection prevalence in F0 is therefore reported descriptively. For F1, a binomial GLM with a logit link was fitted, with Firth penalised likelihood applied.

Total number of mature spore load was quantified for confirmed infected animals, including those with zero mature spores, where infection was established but sporulation failed. We define mature spores as needle‐shaped ascospores enclosed within fully developed asci, the stage responsible for horizontal transmission of *Australozyma*. Sporulation refers to the terminal stage of within‐host development during which vegetative cells that have proliferated by budding in the haemolymph differentiate into mature asci containing the ascospores. Thus, sporulation failure denotes an established infection that did not progress to the production of transmissible asci. Zero spore counts were therefore treated as genuine biological outcomes rather than missing data.

For F0, statistical modelling of mature spore load was not performed because only two nanoplastic‐exposed infected individuals remain after applying the sequential inclusion criteria, resulting in insufficient sample size for reliable parameter estimation. For F1, a Gaussian GLM was fitted initially but failed to converge due to insufficient sample size relative to within‐group variance. Mature spore load is therefore reported descriptively for both generations, including group means, standard deviations, medians and observed ranges, together with individual data points.

## 3 Results

### 3.1 Effects of Direct Versus Maternal Nanoplastic Exposure on Host Fitness

#### 3.1.1 Offspring Production

All offspring produced were female. In F0, direct nanoplastic exposure reduced total offspring production by 95%, and parasite‐inoculated individuals produced fewer offspring than non‐inoculated ones (Figure 2A, Table 1A). No interaction between nanoplastic and parasite treatments (Table 1A). In the F1, offspring of nanoplastic‐exposed mothers (F1‐NP/maternal nanoplastic exposed) produced 58% fewer offspring than offspring of control mothers (F1‐C) (Figure 2A, Table 1A). Neither parasite treatment nor interaction with maternal nanoplastic exposure affected offspring production (Table 1A).

**FIGURE 2:**
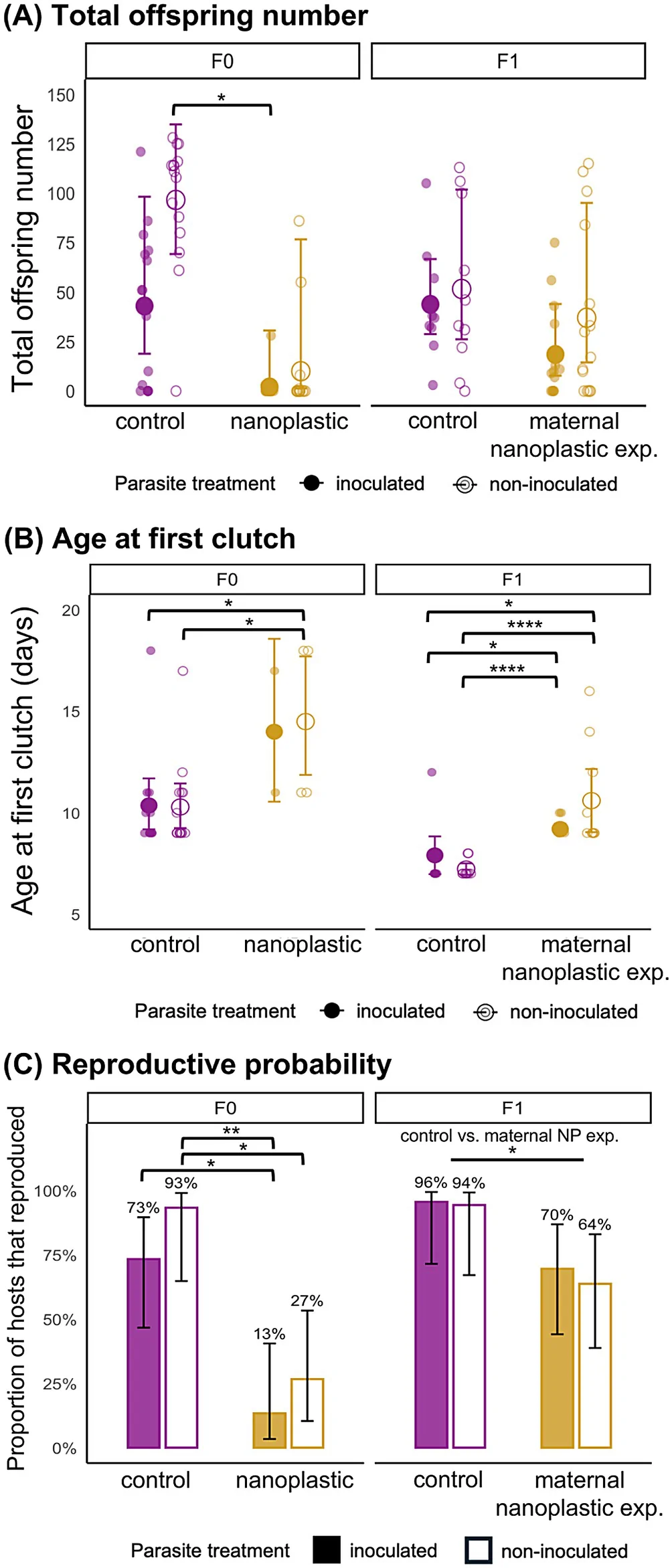
Host fitness parameters in F0 (direct nanoplastic exposure) and F1 (maternal nanoplastic exposure) *Daphnia magna* across nanoplastic and parasite inoculation treatments. Colours indicate nanoplastic treatment (control = dark magenta, nanoplastic (NP) = goldenrod); filled symbols and bars = inoculated, open = non‐inoculated. Large points show model‐estimated marginal means with 95% confidence intervals; individual points are jittered raw data. (A) Total offspring number, from negative binomial GLMs with group‐specific dispersion. (B) Age at first clutch (days), restricted to reproducing individuals; F0 estimates from a Gamma GLM, F1 from a Gaussian GLM with group‐specific dispersion. (C) Reproductive probability (probability of reproducing at least once). Bars show model‐estimated probabilities from binomial GLMs, with percentages giving each group's estimated probability. For F1, the full interaction model produced complete separation, so an additive model with Firth penalised likelihood was used, and the significant pairwise comparison shown (control versus maternal nanoplastic exposure) is based on that model. Horizontal bars show significant Tukey‐adjusted pairwise comparisons between estimated marginal means. Group comparison (control versus maternal nanoplastic exposure) was performed for F1 generation in (C), based on the Firth additive model due to separation. Asterisks denote significance levels: *p < 0.05, **p < 0.01, ***p < 0.001, ****p < 0.0001.

**TABLE 1:** Model estimates for differences in host reproductive fitness parameters in F0 ( *Daphnia magna* directly exposed to nanoplastics) and F1 (offspring of nanoplastic‐exposed mothers raised in nanoplastic‐free conditions).

(A) Negative binomial model estimates for total offspring number				
**F0**				
**AIC**	**BIC**	**logLik**	−2*log(*L*)	**df. resid**.
359.2	412.0	−189.6	379.2	52
**Conditional model**	**Estimate**	**Std. err**.	***z*‐value**	**Pr(> |*z*|)**
(Intercept)	3.761	0.422	8.924	< 2e‐16
**NP treatment**	−3.035	1.439	−2.110	0.0349*
Parasite treatment	0.809	0.454	1.781	0.0749
NP × parasite treatment	0.767	1.783	0.430	0.6669
**F1**				
**AIC**	**BIC**	**logLik**	−2*log(*L*)	**df. resid**.
443.8	459.1	−213.9	427.8	42
**Conditional model**	**Estimate**	**Std. err**.	***z*‐value**	**Pr(> |*z*|)**
(Intercept)	3.780	0.215	17.605	< 2e‐16
NP treatment	−0.864	0.492	−1.755	0.0792
Parasite treatment	0.164	0.408	0.402	0.6879
NP × parasite treatment	0.533	1.770	0.692	0.4891

(B) Model estimates for age at first clutch				
**F0 (Gamma)**				
**AIC**	**BIC**	**logLik**	−2*log(*L*)	**df. resid**.
147.3	154.5	−68.7	137.3	26
Dispersion estimate for Gamma family (sigma^2^): 0.0418				
**Conditional model**	**Estimate**	**Std. err**.	***z*‐value**	**Pr(> |*z*|)**
(Intercept)	2.338	0.062	37.94	< 2e‐16
NP treatment	0.301	0.157	1.91	0.0556
Parasite treatment	−0.008	0.082	−0.09	0.9270
NP × parasite treatment	0.043	0.195	0.22	0.8271
**F1 (Gaussian)**				
**AIC**	**BIC**	**logLik**	−2*log(*L*)	**df. resid**.
117.6	130.9	−50.8	101.6	31
**Conditional model**	**Estimate**	**Std. err.**	***z*‐value**	**Pr(> |z|)**
(Intercept)	7.900	0.457	17.280	< 2e‐16
**NP treatment**	1.300	0.474	2.741	0.00613**
Parasite treatment	−0.678	0.478	−1.419	0.15595
**NP × parasite treatment**	2.078	0.910	2.283	0.02242*

(C) Binomial model estimates for reproductive probability				
**F0**				
**AIC**	**BIC**	**logLik**	−2*log(*L*)	**df. resid**.
61.9	70.3	−27.0	53.9	56
**Conditional model**	**Estimate**	**Std. err.**	***z*‐value**	**Pr(> |*z*|)**
(Intercept)	1.012	0.584	1.733	0.08317
**NP treatment**	−2.883	0.958	−3.010	0.00262**
Parasite treatment	1.628	1.188	1.369	0.17086
NP × parasite treatment	−0.767	1.527	−0.503	0.61523
**F1**				
**AIC**	**BIC**	**logLik**	−2*log(*L*)	**df. resid**.
52.0	57.7	−23.0	46.0	47
**Conditional model**	**Estimate**	**Std. err**.	***z*‐value**	**Pr(> |*z*|)**
(Intercept)	3.084	1.104	2.793	0.00522
**NP treatment**	−2.256	1.098	−2.055	0.03983*
Parasite treatment	−0.263	0.727	−0.362	0.71741

*Note:* Estimates quantify the effect of each factor and their interaction on: (A) total offspring number, negative binomial GLM with group‐specific dispersion; (B) age at first clutch, Gamma GLM with log link (F0) and Gaussian GLM with group‐specific dispersion (F1); (C) reproductive probability, binomial GLM with logit link, with Firth penalised likelihood applied for F1 (logistf). Significant factors are highlighted in bold. Significance codes: *p < 0.05, **p < 0.01, ***p < 0.001, ****p < 0.0001.

#### 3.1.2 Age at First Clutch

Among F0 individuals that reproduced, nanoplastic‐exposed ones produced their first clutch approximately 4 days later than controls (Figure 2B, Table 1B). This estimate is conservative, as most nanoplastic‐exposed individuals failed to reproduce. Neither parasite treatment nor the nanoplastic and parasite interaction affected the age at first reproduction (Table 1B). In F1, maternal nanoplastic exposure delayed reproductive onset, with the magnitude of the delay depending on parasite treatment, as indicated by a significant nanoplastic and parasite interaction (Figure 2B, Table 1B). Among inoculated individuals, F1‐NP reproduced 1.3 days later than F1‐C, whereas among non‐inoculated individuals the delay was 3.4 days. Parasite treatment alone did not affect reproductive timing (Table 1B).

#### 3.1.3 Reproductive Probability

In F0, direct nanoplastic exposure reduced the probability of reproduction by about 82% among parasite‐inoculated individuals and 71% in non‐inoculated ones (Figure 2C, Table 1C). The similarity of these reductions is consistent with the absence of a significant parasite effect or nanoplastic and parasite interaction (Table 1C). Maternal nanoplastic exposure also reduced reproductive probability in F1, with F1‐NP individuals showing roughly 27% lower reproductive odds than F1‐C among parasite‐inoculated individuals and 33% lower odds among non‐inoculated ones (Figure 2C, Table 1C). Parasite treatment had no effect on reproductive probability (Table 1C).

#### 3.1.4 Host Survival

Nanoplastic exposure had a pronounced effect on host survival in the F0 generation. Survival differed among treatment groups by the log‐rank test, and AFT models (Weibull) confirmed that nanoplastic‐exposed individuals died sooner than controls (Figure 3, Table 2A). Non‐inoculated individuals survived longer than parasite‐inoculated ones, suggesting a potential additive mortality cost of nanoplastic exposure and parasite inoculation. Median survival was 9 days in parasite‐inoculated F0‐NP individuals and 10 days in non‐inoculated F0‐NP, compared with 21 days in parasite‐inoculated F0‐C. Median survival was not reached in the F0‐C non‐inoculated group, where 11 of 15 individuals survived to day 30. In the F1, survival did not differ among treatment groups (Figure 3, Table 2B). Neither maternal nanoplastic exposure, parasite treatment, nor their interaction influenced survival time (Table 2B). Median survival ranged from 13 to 17 days across groups, with no clear separation between F1‐NP and F1‐C.

**FIGURE 3:**
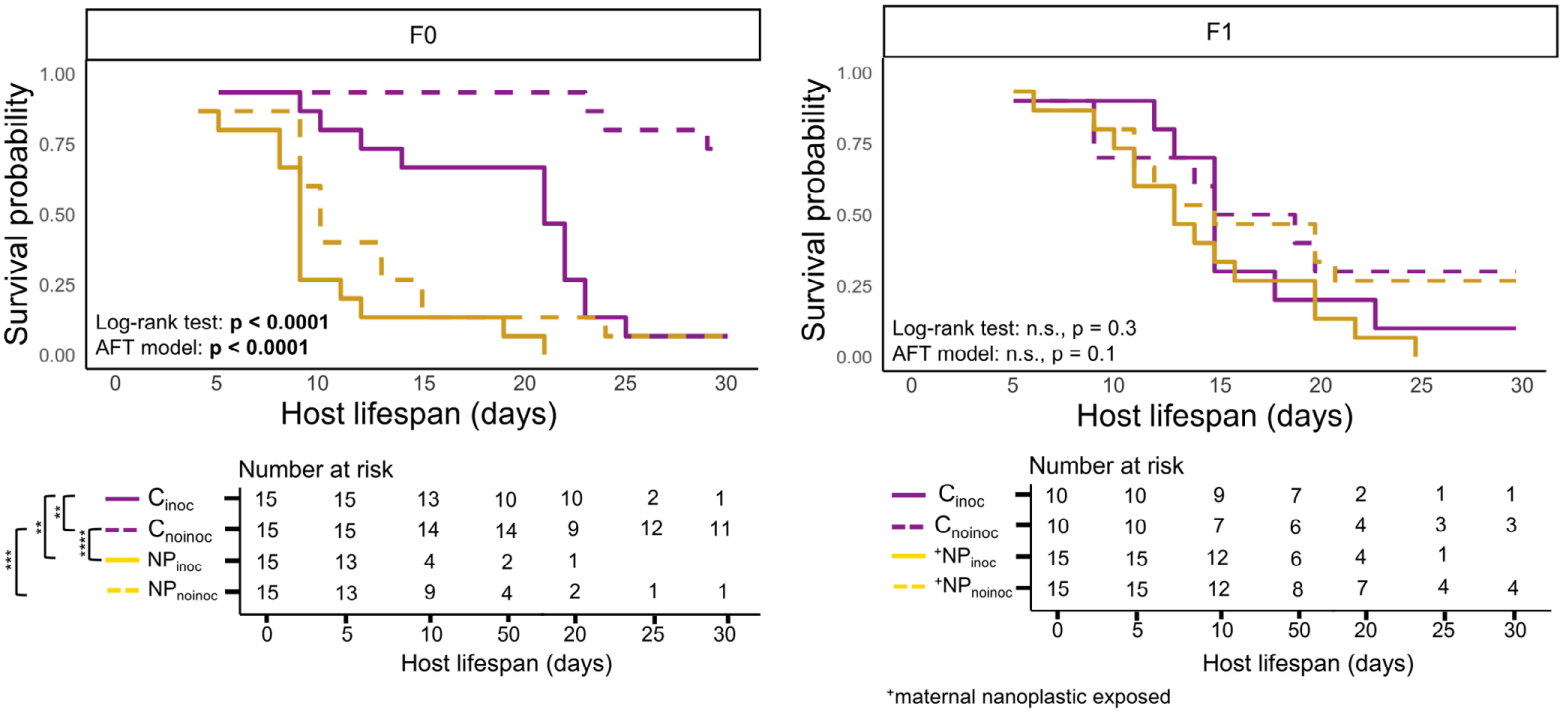
Kaplan–Meier curves for F0 (direct nanoplastic exposure) and F1 (maternal nanoplastic exposure) *Daphnia magna* over a 30‐day experimental period. Death was the event of interest (event = 1); animals alive at day 30 were right‐censored (event = 0). Curves are shown separately for each treatment group combination. C = control; NP = nanoplastic exposure (F0) or maternal nanoplastic exposed (F1); subscripts for parasite treatment: inoc = inoculated, noinoc = non‐inoculated. Line colour indicates nanoplastic treatment (control = dark magenta, NP = goldenrod); line type indicates parasite inoculation treatment (solid = inoculated, dashed = non‐inoculated). Numbers at risk at days 0, 5, 10, 15, 20, 25 and 30 are shown in the risk table below each panel. Group differences were assessed with the log‐rank test, and the effects of nanoplastic and parasite treatment, and their interaction on survival time were estimated from Accelerated Failure Time (AFT) models with a Weibull distribution fitted separately per generation. Horizontal bars show significant Tukey‐adjusted pairwise comparisons between estimated marginal means from the Weibull AFT model. Asterisks denote significance levels: *p < 0.05, **p < 0.01, ***p < 0.001, ****p < 0.0001.

**TABLE 2:** Differences in survival between treatment groups for (A) F0 ( *Daphnia magna* directly exposed to nanoplastics) and (B) F1 (offspring of nanoplastic‐exposed mothers raised in nanoplastic‐free conditions) by treatment group.

(A) Log‐rank test and AFT model estimates for survival in F0					
Log‐rank test (*χ* ^2^ = 42.9, df = 3, **** ***p* = 3e‐09**)					
**Group**	***N***	**Observed**	**Expected**	**(O–E)** ^**2**^ **/E**	**(O–E)** ^**2**^ **/V**
*C*_inoc_	15	14	13.14	0.056	0.0875
*C*_noinoc_	15	4	19.87	12.676	27.5480
NP_inoc_	15	15	5.52	16.298	22.3017
NP_noinoc_	15	14	8.47	3.610	5.0526
AFT model (*χ* ^2^ = 50.13, df = 3, **** ***p* = 7.5e‐11**)					
		**Value**	**Std. err**.	***z*‐value**	***p***
(Intercept)		3.028	0.123	24.67	< 2e‐16
**NP treatment**		−0.634	0.171	−3.71	0.00021***
**Parasite treatment**		0.918	0.270	3.40	0.00067***
NP × parasite treatment		−0.622	0.113	−1.95	0.05162
Loglik(model) = −161.4, Loglik(intercept only) = −186.5					
Number of Newton‐Rhapson iterations = 6, *n* = 60					

(B) Log‐rank test and AFT model estimates for survival in F1					
Log‐rank test (*χ* ^2^ = 3.5, df = 3, *p* = 0.3)					
**Group**	***N***	**Observed**	**Expected**	**(O–E)** ^**2**^ **/E**	**(O–E)** ^**2**^ **/V**
*C*_inoc_	10	9	8.59	0.020	0.0279
*C*_noinoc_	10	7	9.30	0.567	0.8144
NP_inoc_	15	15	10.47	1.964	2.9425
NP_noinoc_	15	11	13.65	0.514	0.8571
AFT model (*χ* ^2^ = 6.16, df = 3, *p* = 0.1)					
		**Value**	**Std. err**.	***z*‐value**	***p***
(Intercept)		2.905	0.163	17.83	< 2e‐16
NP treatment		−0.189	0.206	−0.92	0.36
Parasite treatment		0.278	0.246	1.13	0.26
NP × parasite treatment		−0.718	0.313	−5.67	0.66
Loglik(model) = −153.5, Loglik(intercept only) = −156.6					
Number of Newton‐Rhapson iterations = 5, *n* = 50					

*Note:* C = control; NP = nanoplastic exposure (F0) or maternal nanoplastic exposed (F1); Subscripts: inoc = inoculated; noinoc = non‐inoculated. Overall group differences were assessed using the log‐rank test. Differences in survival time attributable to nanoplastic (NP) treatment, parasite treatment and their interaction were estimated from Accelerated Failure Time (AFT) models with a Weibull distribution fitted separately per generation. Significant factors are highlighted in bold. Significance codes: *p < 0.05, **p < 0.01, ***p < 0.001, ****p < 0.0001.

### 3.2 Effects of Direct Versus Maternal Nanoplastic Exposure on Parasite Fitness

#### 3.2.1 Pre‐Transmission Survival

Nanoplastic exposure drastically reduced host pre‐transmission survival in F0 (Figure 4A, Table 3A). Only 13.3% of F0‐NP individuals survived long enough for the parasite to reach the transmissible stage, compared with 73.3% of F0‐C individuals. In F1, maternal nanoplastic exposure has no effect on pre‐transmission survival (Figure 4A, Table 3A), although the descriptive pattern was similar: 60.0% of F1‐NP individuals survived to the transmissible stage compared with 80.0% of F1‐C individuals (Table 3A).

**FIGURE 4:**
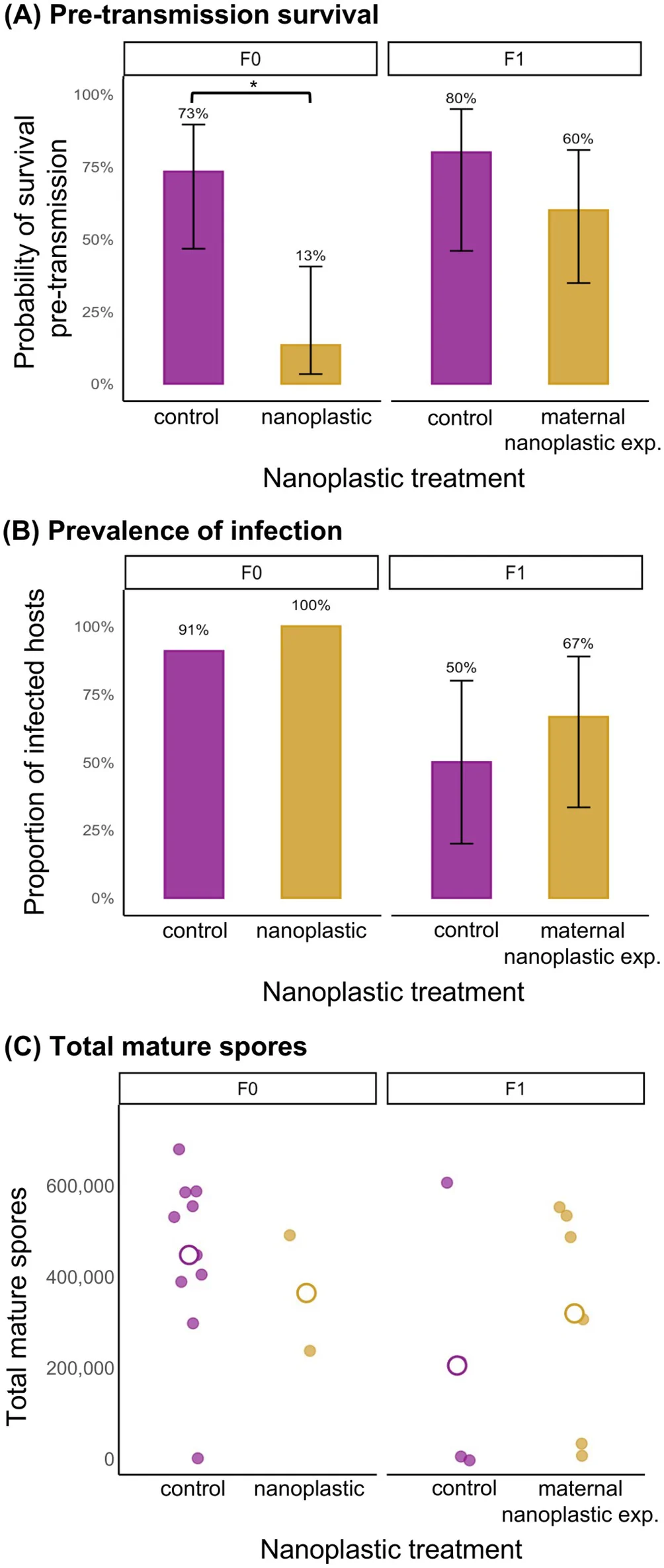
Parasite fitness parameters in F0 (direct nanoplastic exposure) and F1 (maternal nanoplastic exposure) *Daphnia magna* across nanoplastic treatment groups. Colours indicate nanoplastic (NP) treatment (control = dark magenta, NP = goldenrod); percentages indicate group estimates. (A) Pre‐transmission survival probability in inoculated animals, defined as surviving long enough for the parasite to reach the transmissible stage. Bars show model‐estimated probabilities from binomial GLMs with 95% confidence intervals, and percentages give each group's estimated probability. (B) Prevalence of infection in animals surviving to the pre‐transmission stage. F0 is descriptively only due to insufficient survivors (*n* = 2) in the nanoplastic treatment group, and no confidence intervals are shown; F1 bars show model‐estimated probabilities from a binomial GLM with Firth penalised likelihood. (C) Total mature spores in confirmed infected animals. Individual points are shown with group means marked separately. Zero counts indicate hosts in which infection established but no mature spores were produced (sporulation failure). No model was fitted due to small sample sizes and model convergence failure in F1, so results are descriptive only. Horizontal bar in (A) show a significant difference between control and nanoplastic treatments (binomial GLM). Asterisks denote significance levels: *p < 0.05, **p < 0.01, ***p < 0.001, ****p < 0.0001.

**TABLE 3:** Differences in parasite fitness parameters between nanoplastic (NP) and control treatments among inoculated F0 ( *Daphnia magna* directly exposed to nanoplastics) and F1 (offspring of nanoplastic‐exposed mothers raised in nanoplastic‐free conditions).

(A) Binomial model estimates for pre‐transmission survival				
**F0**				
**AIC**	**BIC**	**logLik**	−2*log(*L*)	**df. resid**.
33.2	36.0	−14.6	29.2	56
**Conditional model**	**Estimate**	**Std. err**.	***z*‐value**	**Pr(> |*z*|)**
(Intercept)	1.012	0.584	1.733	0.08317
**NP treatment**	−2.883	1.950	−3.010	0.00262**
**F1**				
**AIC**	**BIC**	**logLik**	−2*log(*L*)	**df. resid**.
34.2	36.6	−15.1	30.2	23
**Conditional model**	**Estimate**	**Std. err**.	***z*‐value**	**Pr(> |*z*|)**
(Intercept)	1.386	0.791	1.754	0.0795
NP treatment	−0.981	0.950	−1.032	0.3019

(B) Descriptive summary (F0) and binomial model estimate (F1) for prevalence of infection			
**F0**			
**NP treatment**	***n***	**Infected**	**Prevalence (%)**
Control	11	10	90.9
NP	2	2	100

**F1**						
	**Coef**	**Std. err.**	**Lower 0.95**	**Upper 0.95**	***χ* ^2^**	***p***
(Intercept)	−7.8e‐17	0.667	−1.354	1.354	0.000	1.000
NP treatment	6.2e‐01	0.940	−1.216	2.540	0.438	0.508
Method: Profile penalised log‐likelihood method, likelihood ratio test = 0.438, df = 1, *p* = 0.508, *n* = 17						

(C) Descriptive summary for total mature spores					
**F0**					
**NP treatment**	***n***	**Zeros**	**Mean ± std. dev**.	**Median**	**Range**
Control	10	1	452,494 ± 196,639	484,875	0–687,844
NP	2	0	367,688 ± 181,505	367,688	239,344–496,031
**F1**					
**NP treatment**	***n***	**Zeros**	**Mean ± std. dev**.	**Median**	**Range**
Control	4	2	205,992 ± 288,787	106,203	0–612,562
NP	6	1	322,220 ± 254,295	311,719	0–558,938

*Note:* Estimates compare NP and control groups for: (A) pre‐transmission survival probability from binomial GLMs with a logit link; (B) prevalence of infection among hosts surviving to the pre‐transmission stage, presented descriptively for F0 due to insufficient NP survivors (*n* = 2) and estimated from a binomial GLM with Firth penalised likelihood for F1; (C) total mature spores descriptive summary in confirmed infected hosts, including hosts in which infection established but sporulation failed (total mature spore count = 0). No statistical model was fitted for (C) in either generation due to small sample sizes after sequential biological filtering or model convergence failure in F1. Values shown are group means ± standard deviation and observed ranges. Significance codes: *p < 0.05, **p < 0.01, ***p < 0.001, ****p < 0.0001.

#### 3.2.2 Prevalence of Infection

Infection prevalence among F0 individuals surviving to the pre‐transmission stage was high in both groups: 10 of 11 F0‐C individuals (90.9%) and both surviving F0‐NP individuals (100%) were infected. The small number of surviving F0‐NP hosts (*n* = 2) precluded statistical comparison; therefore, infection prevalence is reported descriptively only (Figure 4B, Table 3B). In F1, infection prevalence did not differ between F1‐NP (50%) and F1‐C individuals (67%) (Figure 4B, Table 3B).

#### 3.2.3 Mature Spore Production

Total mature spore production is reported descriptively because high mortality resulted in small sample sizes. In F0, 11 of 12 infected individuals produced mature spores. Mean mature spore production was 452,494 ± 196,639 spores in F0‐C and 367,688 ± 181,505 spores in F0‐NP, with the latter estimate on only two individuals. In F1, 7 of 10 infected individuals produced mature spores, with mean production of 205,992 ± 288,787 spores in F1‐C and 322,220 ± 254,295 spores in F1‐NP (Figure 4C, Table 3C). Given the small sample sizes and high variability, these descriptive differences should be interpreted cautiously.

## 4 Discussion

Here, we investigated the direct effects of nanoplastic exposure on host and parasite fitness in the parental generation (F0) and assessed whether these effects carried over to unexposed F1 offspring through maternal effects. Consistent with expectations, nanoplastic exposure in F0 strongly reduced host fitness across reproductive and survival traits. This was accompanied by reduced pre‐transmission survival following parasite exposure, indicating that nanoplastic‐induced host mortality constrains the window for parasite maturation. However, among hosts that survived to the transmissible stage, infection prevalence and spore production showed no consistent evidence of enhanced within‐host parasite performance. In the F1 generation, maternal nanoplastic exposure generated clear transgenerational effects on host reproduction, whereas parasite‐related outcomes remained largely unaffected. Together, these findings demonstrate that nanoplastic exposure disrupts host–parasite interactions primarily through direct and maternal effects on host performance, with comparatively weaker effects on parasite development within surviving infections.

### 4.1 Direct Nanoplastic Exposure Strongly Impairs Host Fitness

The near‐complete reproductive failure and dramatically shortened survival of directly exposed F0 individuals reflect a systemic physiological collapse rather than isolated trait‐specific effects, consistent with previous reports of nanoplastic‐induced fitness impairment in *Daphnia* (Antonio Vital et al. 2025; Kelpsiene et al. 2020; Liu et al. 2019). These broad fitness costs likely arise from multiple interacting physiological pathways. Nanoplastic ingestion reduces energy intake by impairing filter‐feeding efficiency (Rist et al. 2017) while increasing metabolic demands through oxidative stress responses (Lee et al. 2024; Liao et al. 2023), inflammatory activation (Liao et al. 2022) and microbiome disruption (Villegas et al. 2025). Together, these processes likely shift allocation away from reproduction toward cellular repair and stress responses, consistent with dysregulation of reproductive metabolic processes under nanoplastic exposure (Liu et al. 2021; Zhang et al. 2020). Nanoplastic exposure has been shown to downregulate chitinase gene expression in *Daphnia* (Zhang et al. 2020), potentially delaying moulting and consequently reproductive onset as moulting is a prerequisite for neonate release.

Nanoplastic effects on *Daphnia* are strongly size‐ and dose‐dependent (Han et al. 2024; Liu, Liao, et al. 2024). The pronounced effects observed here are consistent with the use of a very small particle size (50 nm). Smaller nanoplastics generally elicit stronger toxicity than larger particles (Liu, Liao, et al. 2024; Meng et al. 2023), likely due to higher surface‐area‐to‐volume ratios that enhance cellular uptake and subcellular interactions (Gigault et al. 2021; reviewed in Pochelon et al. 2021; Tang 2025). This underscores that particle size is a key determinant of nanoplastic biological impact that should be carefully considered in comparative studies.

### 4.2 Parasite Exposure Imposes Independent Costs on Host Fitness

Parasite exposure imposed additional costs on host fitness in F0, that were largely independent of nanoplastic treatment, with no evidence for a statistical interaction between the two stressors. This suggests additive rather than synergistic effects under the conditions tested. Nanoplastic exposure can induce oxidative stress and increase metabolic demand (Lee et al. 2024; Liao et al. 2023), while infection imposes additional energetic cost on the host (Fearon et al. 2025; Paraskevopoulou et al. 2026). The lack of statistical interaction might therefore indicate that nanoplastic exposure and infection act through at least partially distinct physiological pathways.

The comparatively weak effect of parasite inoculation on host reproduction, particularly in F1, may partly reflect the definition of the inoculated group in the host fitness analyses. All inoculated individuals were included irrespective of whether infection successfully established, such that the inoculated group comprised both infected and uninfected hosts. Since uninfected hosts were likely to maintain higher fitness than confirmed infected hosts, potentially approaching that of non‐inoculated controls, their inclusion would reduce the contrast between inoculated and non‐inoculated groups and thereby dilute the estimated effect of parasite exposure. This dilution was likely greater in F1, where only 50% of inoculated control individuals became infected, compared with 91% in F0. Thus, differences in infection prevalence between generations may have contributed to the weaker apparent effect of parasite inoculation on F1 host fitness, rather than an indication that parasite infection itself imposed weaker costs on F1 hosts.

The lower prevalence in F1 may, in turn, reflect differences in offspring susceptibility arising from maternal or rearing conditions. In *Daphnia*, susceptibility to infection can be influenced by offspring phenotype, including body size and feeding rate, with larger offspring generally being less susceptible (Garbutt and Little 2017). Maternal condition can also substantially alter offspring susceptibility, with offspring produced under poorer maternal conditions showing more than a twofold reduction in susceptibility in some contexts (Mitchell and Read 2005). Although these traits were not assessed as mechanisms in the present study, such differences could contribute to the lower infection prevalence observed in F1. More broadly, maternal nanoplastic exposure might have altered offspring responses to parasite challenge by modifying either resistance to infection or tolerance of its fitness consequences. Maternal parasite exposure, for example, can modify offspring life‐history traits and increase reproduction under infection in *Daphnia magna* (Paraskevopoulou et al. 2022), although such effects are strongly context‐dependent and can also reduce offspring fitness (McIntire et al. 2025). Consequently, the reduced prevalence and weaker apparent parasite effects in F1 should not necessarily be interpreted as a simple attenuation of parasite‐induced costs but may instead reflect differences in offspring susceptibility and maternal carry‐over effects.

### 4.3 Maternal Nanoplastic Exposure Causes Persistent but Trait‐Specific Transgenerational Reproductive Impairment

The recovery of survival alongside persistent reproductive impairment in F1 offspring of nanoplastic‐exposed mothers is the most striking pattern in the transgenerational traits. Reproductive timing and reproductive probability remained compromised in offspring of nanoplastic‐exposed mothers, whereas survival was fully restored, pointing to differential sensitivity of life‐history traits to maternal nanoplastic exposure. This pattern is consistent with a maintenance‐fecundity trade‐off (He et al. 2023; Nogueira et al. 2022) in which nanoplastic‐stressed mothers might prioritise somatic investment at the expense of reproductive provisioning, transmitting a reproductive but not a survival deficit to offspring. The reduction in total offspring production under maternal nanoplastic exposure in F1 reinforces this putative mechanism, suggesting the reproductive legacy of maternal nanoplastic exposure extends across multiple fecundity metrics.

The significant interaction between maternal nanoplastic history and F1 inoculation status for age at first clutch indicates that infection partially masks the maternal nanoplastic effect on reproductive timing. Non‐inoculated F1‐NP individuals exhibited the full delay in reproduction, while inoculated F1‐NP showed a smaller delay. One possible explanation is a terminal investment response, whereby increased infection‐induced mortality risk accelerates reproduction regardless of maternal exposure history (Clutton‐Brock et al. 1984). Although our data cannot distinguish this mechanism from other effects of infection on reproductive timing, the interaction suggests that maternal nanoplastic effects on reproduction may be most pronounced in the absence of infection pressure. The timing of first reproduction is a key driver of the intrinsic rate of population increase in *Daphnia* (Coors and De Meester 2008), suggesting that delays induced by maternal nanoplastic exposure could reduce population growth under conditions of low infection pressure. Given the key role of *Daphnia* as a keystone grazing organism linking individual life‐history variation to community and ecosystem processes (Miner et al. 2012), transgenerational shifts in reproductive timing could ultimately influence zooplankton community structure and top‐down control of phytoplankton.

The shift from near‐complete reproductive failure in F0‐NP individuals to a more moderate reduction in reproduction in F1‐NP indicates attenuation of the transgenerational signal, consistent with partial epigenetic inheritance (Danchin et al. 2011; Mousseau and Fox 1998) and previous studies showing that maternal exposure to micro‐ and nanoplastics reduces offspring reproduction in *Daphnia* (Liao et al. 2023; Martins and Guilhermino 2018). More broadly, this finding illustrates that both the magnitude and persistence of maternal effects depend on the trait considered and the environmental context experienced by the offspring. *In Daphnia*, maternal environments shaped by predation risk and resource limitation (reviewed in Agrelius and Dudycha 2025), food quality‐temperature interactions (Pajk et al. 2012) and elevated temperature combined with parasitic infection (Sun et al. 2023), can all influence offspring life‐history traits. Together, these findings emphasise that maternal effects are not necessarily uniform across offspring traits or environmental conditions, and that their phenotypic consequences may be modified by subsequent ecological stressors such as parasite infection.

### 4.4 Nanoplastic Exposure Constrains Parasite Fitness Primarily Through Host Mortality

The near‐elimination of pre‐transmission survival in directly exposed F0 hosts reveals that nanoplastic exposure can effectively reduce the pool of hosts available for parasite transmission, not by increasing resistance, but through host mortality before the parasite reaches the transmissible stage. This distinction is epidemiologically important because contaminants that increase host mortality have fundamentally different consequences for disease dynamics than those that primarily affect host immunity and resistance (Cressler et al. 2016; Stewart Merrill and Johnson 2020). Functionally, mortality occurring before parasite maturation acts as a sink for the parasite by removing infected hosts before they contribute to the transmission. Such reductions in host availability are predicted to decrease parasite prevalence consistent with the healthy herds hypothesis (Packer et al. 2003) and have been demonstrated in the *Daphnia dentifera–Australozyma monospora* system, where predation reduced prevalence while simultaneously depleting host density (Lopez et al. 2023). Thus, pollutant‐driven host mortality could potentially suppress epidemics by reducing the number of hosts reaching the infectious stage, although this effect is not universal. Non‐selective mortality that releases spores can amplify environmental transmission and, consequently, disease dynamics (Cáceres et al. 2009).

Among surviving nanoplastic‐exposed hosts that survived to the transmissible stage, infection prevalence remained high and mature spore production showed no obvious differences from controls, suggesting that parasite development was not strongly impaired. Rather than directly reducing parasite performance within hosts, nanoplastic exposure constrained parasite fitness primarily by reducing host survival to the transmittable stage. At the concentration used here, host mortality dominated the outcome of host–parasite interaction and severely reduced parasite maturation. At lower, sub‐lethal concentrations, the balance between host mortality and within‐host parasite performance might shift, potentially allowing parasites to persist in longer‐lived hosts even if nanoplastic exposure compromises host condition or immune function.

In F1, offspring of nanoplastic‐exposed mothers showed lower pre‐transmission survival and higher spore loads, but neither effect was statistically supported. If F1‐NP animals are subtly immunocompromised, this would represent a particularly striking transgenerational outcome: offspring could be simultaneously reproductively impaired and more permissive to parasite development. This pattern, where immunocompromised hosts support higher parasite burdens, has precedent in *Daphnia*‐parasite systems (Auld et al. 2012) and warrants direct experimental investigation with larger, family‐tracked cohorts designed to adequately power parasite fitness comparisons across generations.

### 4.5 Possible Underlying Mechanisms, Limitations and Future Directions

Several mechanisms may underlie the observed maternal effects. Stress‐induced changes in maternal energetic state (reviewed in Agrelius and Dudycha 2025) and resource allocation to reproduction (Harney et al. 2017; Wacker and Martin‐Creuzburg 2007) can alter offspring performance even when offspring develop in contaminant‐ and stress‐free conditions. Epigenetic regulation through DNA methylation, histone modification or RNA‐mediated gene silencing represents another plausible pathway (Fitz‐James and Cavalli 2022; Klibaner‐Schiff et al. 2024), with environmentally induced methylation changes shown to persist across generations in *Daphnia* (Feiner et al. 2022). Additional mechanisms include direct transfer of nanoplastics from mother to offspring via the brood chamber and gut‐ovary‐oocyte pathways (Xue et al. 2023), and microbiome alteration caused by nanoplastic exposure (Villegas et al. 2025, 2026) which can be inherited by the offspring. Disentangling these pathways would substantially advance understanding of how nanoplastic maternal effects are transmitted and expressed.

Several aspects of the experimental design should be considered in future studies. First, the pooled F1 design prevented quantification of among‐mother variation, limiting inference about heterogeneity in maternal effects. Future studies should therefore track maternal families explicitly, allowing quantification of maternal variance components and more precise estimation of transgenerational effect sizes. Second, dose–response designs would help determine how the magnitude of maternal effects varies with exposure intensity, disentangle the independent contributions of particle size and concentration, and test whether these effects persist at environmentally relevant, sub‐lethal concentrations. Finally, extending observation to F2 and subsequent generations would help determine whether the reproductive impairment observed here continues to attenuate or instead stabilises as a persistent lineage effect.

## 5 Conclusions

Nanoplastic exposure imposes severe and cascading costs on *Daphnia magna* that extend beyond the directly exposed generation. Direct exposure drives near‐complete reproductive failure, accelerates mortality and constrains parasite maturation through host mortality. These effects partially persist in unexposed offspring through maternal effects, with reproductive timing and reproductive probability remaining compromised while survival recovers. In contrast, parasite‐related outcomes showed little evidence of transgenerational effects. Together, these findings underscore that the ecological consequences of nanoplastic pollution extend beyond direct toxicity, generating persistent transgenerational reproductive costs that may slow host population recovery and alter host–parasite dynamics in nanoplastic‐contaminated freshwater environments.

## Author Contributions

**Vanderville Villegas:** conceptualization (lead), formal analysis (equal), methodology (equal), resources (equal), supervision (equal), visualization (equal), writing – original draft (equal). **Lilith Biere:** conceptualization (equal), formal analysis (equal), investigation (lead), methodology (equal), visualization (equal), writing – original draft (equal). **Kristel F. Sánchez:** formal analysis (equal), writing – review and editing (supporting). **Justyna Wolinska:** conceptualization (equal), funding acquisition (lead), methodology (equal), supervision (equal), writing – review and editing (lead).

## Funding

This work was supported by Leibniz Institute of Freshwater Ecology and Inland Fisheries.

## Conflicts of Interest

The authors declare no conflicts of interest.

## Data Availability

Data and R scripts are available in the Zenodo repository (https://doi.org/10.5281/zenodo.20592232).
